# Nanostructures for the Inhibition of Viral Infections

**DOI:** 10.3390/molecules200814051

**Published:** 2015-08-03

**Authors:** Sabine Szunerits, Alexandre Barras, Manakamana Khanal, Quentin Pagneux, Rabah Boukherroub

**Affiliations:** Institute of Electronics, Microelectronics and Nanotechnology (IEMN), UMR 8520 CNRS, Lille1 University, Avenue Poincaré—BP 60069, 59652 Villeneuve d’Ascq, France; E-Mails: alexandre.barras@iri.univ-lille1.fr (A.B.); manukhl@hotmail.com (M.K.); quentin.pagneux@etudiant.univ-lille1.fr (Q.P.); rabah.boukherroub@univ-lille1.fr (R.B.)

**Keywords:** viral infections, antiviral therapy, nanomaterials

## Abstract

Multivalent interactions are omnipresent in biology and confer biological systems with dramatically enhanced affinities towards different receptors. Such multivalent binding interactions have lately been considered for the development of new therapeutic strategies against bacterial and viral infections. Multivalent polymers, dendrimers, and liposomes have successfully targeted pathogenic interactions. While a high synthetic effort was often needed for the development of such therapeutics, the integration of multiple ligands onto nanostructures turned to be a viable alternative. Particles modified with multiple ligands have the additional advantage of creating a high local concentration of binding molecules. This review article will summarize the different nanoparticle-based approaches currently available for the treatment of viral infections.

## 1. Introduction

The constant emergence of new viruses with a global impact to public health and society at large together with the general absence of availability of specific antiviral therapeutics for a variety of viruses have made the search for antiviral drugs and therapeutics a challenging research task. Viruses pose a considerable challenge to the body’s immune system as they hide inside cells, making it difficult for antibodies to reach them. In contrast to bacterial infections, which are mostly treated using antibiotics, immunization against viral infections is not always possible. While vaccines against serious viral infections such as measles, mumps, hepatitis A, and hepatitis B exist, some viruses are capable of mutating from one person to the next, making vaccination a difficult task as the viruses have already changed their format by the time vaccines are available. Over the last 30 years, research has thus focused on the development of new antiviral agents able to positively impact the spread of viral infections. While, by 1990, just five antiviral agents had been licensed [1], more than 40 formulations are currently available on the market. The antiviral therapies currently approved are based on the use of small molecular weight drugs or proteins that stimulate the innate immune response. Table 1 lists some of the most common antiviral agents used in clinical practice. As can be seen, most of these agents were initially developed for the treatment of human immunodeficiency virus (HIV) infections, whereas antiviral drugs against herpes simplex virus (HSV), hepatitis C virus (HCV), and others have emerged in parallel.

**Table 1:** List of some approved antiviral drugs for human immunodeficiency virus (HIV), hepatitis C virus (HCV), herpes simplex virus type 1 and type 2 (HSV-1, HSV-2) [2].

Virus	Antiviral Agent	Administration	Viral Target
**HIV**	Efavirenz, Nevirapine, Rilpivirine	Oral	Non-nucleoside reverse transcriptase inhibitor
	Indinavir		Recombinant protease inhibition
	Raltegravir		Integrase strand transfer inhibitor
	Maraviroc		Antagonists of CCR5 receptor
	Tenofovir		Nucleotide reverse transcriptase inhibitor
	Atazanavir, Ritonavir, Lopinavirb, Ritonavirc, Saquinavir, Tipranavir, Darunavir, Indinavir		Protease inhibitors
	Abacavir, Didanosine, Emtricitabine, Lamivudine		Nucleoside reverse transcriptase inhibitors
	Dapivirine	Intravaginal rings	Non-nucleoside reverse transcriptase inhibitor
	Enfuvirtide	Subcutaneous injection	Inhibitor of gp 41
	Zidovudine	Oral + intravenous	Reverse transcriptase inhibitor and stop DNA elongation
**HCV**	Ribavarin	Oral	Nucleoside analogue
	Pegylated interfereon-α	Subcutaneous	Major histocompatibility complex stimulator
**HSV**	Acyclovir	Oral, topical, intravenous	Inhibitor DNA syntheses
	Penciclovir	Topical	DNA elongation inhibitor
	Famciclovir, Brivudin, Valaciclovir	Oral	DNA elongation inhibitor
	Iodoxuridine	Intravenous	DNA elongation inhibitor
	Trifluridine	Eye drops	DNA elongation inhibitor

While the global market for antiviral drugs is constantly witnessing growth due to the existence of unmet needs and population growth, the development of safe and effective antiviral drugs is a complex and difficult task. Several factors hinder the rapid development of antiviral drugs. Viruses are intracellular parasites and their replication depends on the host-cell biosynthetic machinery. Consequently, only a limited number of virus-specific metabolic functions can be targeted by antiviral agents without harming the host at the same time. Moreover, each virus has specific functions, making the development of broad-spectrum antiviral drugs difficult.

The majority of antiviral drugs are administered orally, although some are delivered via intravenous or subcutaneous routes. The other key challenge to using most of the antiviral drugs is linked to their limited solubility in aqueous media, short half-life time, and/or slow uptake by the body. Taking the example of acyclovir (ACV), a highly efficient drug against herpes viruses: owing to its poor bioavailability following oral dosing (15%–30%), a high level of patient compliance is required, with many patients taking multiple oral doses daily at set times to obtain a relatively constant drug level [3]. As ACV has limited solubility in water (1.62 mg/mL at 22 °C), intramuscular injection or ocular administration is of limited use. Parenteral administration of ACV is presently available as an infusion or as bolus intravenous injections in the form of strong alkaline (pH 10–11) solution, causing, however, pervascular inflammation [4]. Beside the physico-chemical limitations of some drugs, prolonged anti-viral therapy increases, in addition, the likelihood that drug-resistant strains of the virus emerge. Extensive use of ACV has, in particular, resulted in ACV-resistant virus strains in immuno-compromised patients [5].

To improve antiviral treatment, multidisciplinary research efforts are crucial for the development of alternative strategies toward improved antiviral therapeutics. There have been many attempts for improving the physico-chemical properties of antiviral drugs such as acyclovir or vidarabine by chemical modifications [6,7]. Another alternative for the delivery of antiviral drugs is the use of controlled-release delivery vehicles in the form of tablets and patches. Such formulations reduce the administered dose and aim to overcome problems of non-compliance and loss of drug activity. The ideal delivery platform would release the antiviral drug at a constant dose over a long time. Acyclovir delivery vehicles based on silicone polymer were proposed as implants for viable and long-time suppressive therapy of HSV-1 [8].

**Figure 1:**
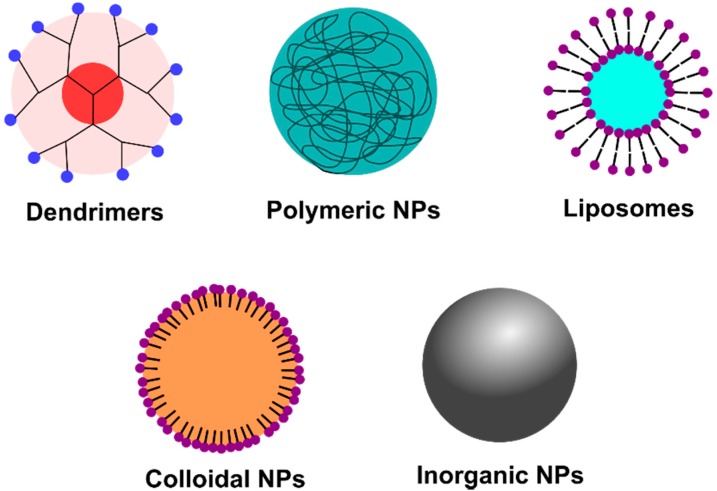
Different types of nanocarriers available for incorporating antiviral agents.

The design of nanomaterials-based delivery systems has several advantages. Nanomaterials have the characteristics of high surface-to-volume ratios, enabling the packaging of multiple antiviral agents onto the same nanoparticles. Using these nanomaterials, it might be possible to overcome problems associated with the use of high doses of antiviral drugs. Moreover, this type of approach also provides the possibility of targeting specific biological sites actively or passively. Because of their unique features, such as nanometric size and controllable hydrophobicity/lipophilicity, such antiviral nanocarriers can target drugs to specific tissues or organs, while the modification of nanocarrier surfaces enables them to reach particular sites to deliver the drug at the specific target. This helps local and systematic delivery of antiviral drugs with the results of minimizing side effects of healthy cells and tissue. With respect to intravenous administration, due to their small size, nanoparticles can circulate in the bloodstream without being retained by the pulmonary capillaries or uptaken by the reticolo-endothelial system (RES). Indeed, the most frequently used approach to increase the longevity of nanocarriers to avoid the RES uptake is to modify their surface with hydrophilic polymers such as polyethylene glycol (PEG) units. Various nanocarriers for antiviral agents have been proposed over the years and some of the most currently considered are listed in Figure 1.

Dendrimers are polymeric nanostructures with unique morphological features. They exhibit three-dimensional tree-like structures connected to a central core [9]. They can be synthesized with precise physico-chemical and biological properties depending on the type of polymer and functional groups used. Due to the presence of numerous surface functions, the conjugation with multiple drugs or targeting ligands to a single dendrimeric structure is possible. While dendrimers have the ability to encapsulate hydrophobic drugs, due to their limited cavity size, the percentage of encapsulation is rather low. One of the most widely known antiviral dendrimeric structure is VivaGel, the first topical nanomicrobicide. VivaGel is a g-4-poly-l-lysine dendrimer formed from the divalent benzhydylamine amide of l-lysine and contains 32 sodium 1-(carboxymethoxy)naphthalene-3,6-disulfonate as terminal anionic functional groups [10].

Polymeric nanoparticles have received great attention for the vaginal delivery of microbicides since the seminal paper by Han *et al.* [11,12]. Polymeric nanoparticles are usually made of biocompatible and/or biodegradable polymers (e.g., alginate, hyaluronic acid, poly(lactic acid), poly(glycolic acid), poly(lactic-co-glycolic acid), polycaprolactone, cyclodextrin, *etc.*) with sizes ranging between 10 and 1000 nm [13,14,15]. The antiviral agent can be encapsulated, adsorbed, conjugated, or dissolved in the polymeric structure [16,17]. The wide choice of polymers allowing sustained release of antiviral agents and improved metabolic stability of the integrated drug have made polymeric particles of great potential for the successful delivery of antiviral agents.

The first developed as well as commercialized nanostructures are, however, liposomes. Liposomes, first described in 1965 by Bangham [18], are vesicular colloidal systems consisting of a bilayer (unilamellar liposomes) or multilayers (multilamellar liposomes) of natural or synthetic phospholipids and an aqueous core. Lipophilic agents can be inserted into the lipid bilayer, while hydrophilic therapeutics can fit into the aqueous compartments. The size of the liposomes can vary between 40 nm and 10 µm, depending upon the preparation method used. The size of the liposome, its surface charge, hydrophobicity, and membrane fluidity also influence encapsulation efficiency, *in vitro* stability, and biodistribution [19]. The tendency of liposomes to get rapidly recognized by phagocytic cells, such as liver and spleen, after intravenous administration can be minimized by chemical modification with poly(ethylene glycol) (PEG) units [20]. The surface of liposomes can be easily engineered with various functions to enhance their recognition as well as uptake by macrophages or other components of the immune system. They are mainly explored for delivering HIV vaccines as well as siRNA [21,22]. While commercially feasible technologies are available for large-scale production, these systems have been shown to aggregate and the active ingredient degraded during storage, thereby reducing the performance of these molecular architectures over long periods of time.

Microemulsions, isotropic systems of oil, water, and a surfactant, are droplets in the nanometer range [23,24]. These thermodynamically stable mixtures allow the entrapment of a wide range of active drugs and have great potential as drug delivery systems. The potential of microemulsions of ACV for transcutaneous administration and suppression of herpetic skin lesions has been demonstrated [25]. Microemulsions can be classified into solid lipid nanoparticles (SLN) and lipid nanocapsules (LNC) [26]. SLN are made of a solid hydrophobic core (triglycerides, fatty acids, steroids) surrounded and stabilized by emulsifiers such as lecithin or polymers [27]. LNCs consist of an oily core surrounded by a crown of triglycerides composed of a lipophilic surfactant such as lecithin and a nonionic one such as Solutol HS15 [28]. The LNC have the advantage of being formulated in the absence of solvents, with biocompatible constituents with a size varying between 20 nm and a few hundred nanometers. Encouraging results on the entrapment of ACV into SLN have been demonstrated [29]. The SLN formulation appeared stable during six months. As the diffusion increased compared to free ACV in solution, it was able to bypass the skin barrier in topical applications.

Finally, inorganic nanoparticles, such as gold, silver, iron oxide, *etc.*, have found their application in antiviral therapy. Elichegurra *et al.* were the first to demonstrate the effect of silver nanoparticles (Ag NPs) on HCV-1 [30]. Several studies showed that Ag NPs interfered with several stages of the viral replication cycle. Penades *et al.* have carried out a series of experiments to explore the multivalent effect of various oligomannosides on Au NPs for the inhibition of dendritic cell-specific intercellular adhesion molecule-3-grabbing non-integrin (DC-SIGN)-mediated HIV transfection. Indeed, as in many biological interactions, the attachment and entry of viruses into the host cells is an outcome of multivalent interactions between the viral surface components and cell membrane receptors [31,32]. Interfering with these recognition events using functional nanoparticles, thereby blocking viral entry into the cells, has been recognized as one of the most promising strategies in the development of new antiviral drugs and approaches.

In this review article, we will discuss in a systematic manner the use of different nanomaterials for the treatment of viral infections (Figure 1). Their advantages and disadvantages will be highlighted using appropriate research publications of the last years. The focus will be on the three most widely investigated viruses responsible for a variety of clinical manifestations: human immunodeficiency virus (HIV), hepatitis C virus (HCV), and Herpes simplex virus type 1 and type-2 (HSV-1 and HSV-2). Some perspectives and a general conclusion will be given at the end of the review. Before discussing the applications of nanomaterials as antiviral agents, a general introduction about the viral replication cycle will be discussed. This will allow non-biologists and non-experts in the field to better understand the different ways that nanomaterials can interfere with viral interactions with host cells.

## 2. Viral Replication Cycle

**Figure 2:**
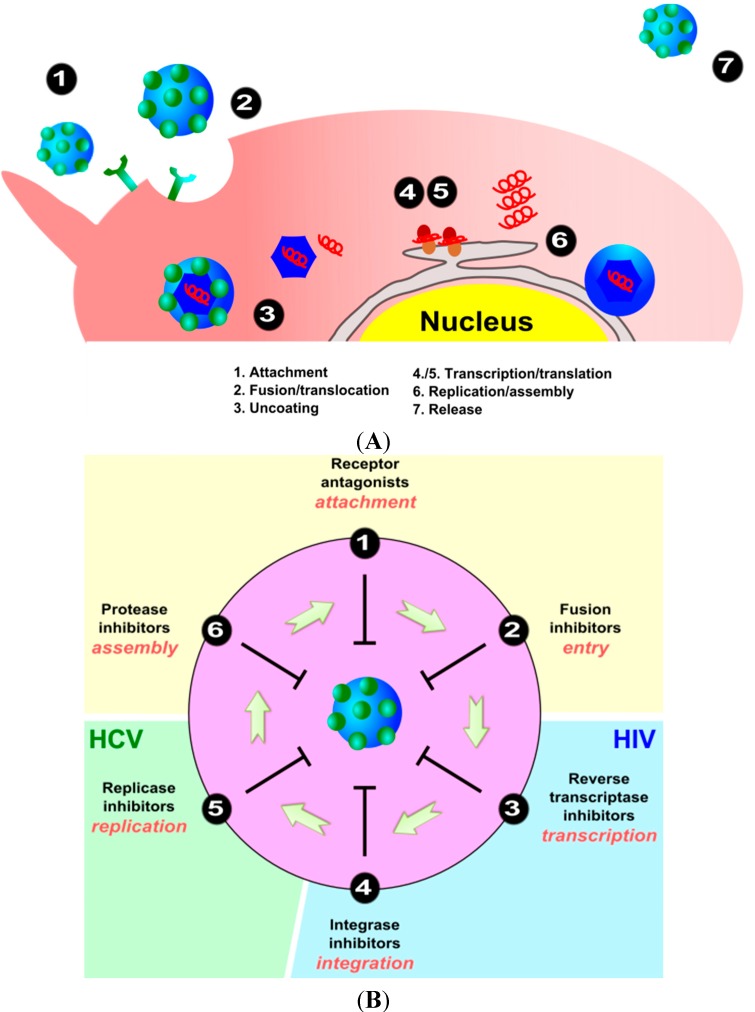
(**A**) Simplified schematic representation of viral replication cycle. Abbreviation ER: endoplasmic reticulum; (**B**) Potential points of attack of antiviral nanostructures in the viral replication cycle.

Viruses are obligate intracellular parasites that carry their genome (RNA or DNA) and some functional proteins and replicate only inside the living cells of organisms, causing the death of the host cell. However, viruses depend on the host cell machinery to complete the viral replication cycle and must lead that machinery to replicate successfully. In each viral replication cycle, 100–1000 new viral particles are produced. All viruses replicate via a broadly similar sequence of events, as highlighted schematically in Figure 2A. The first course of action is the attachment of the surface proteins of the virus to specific receptors on the host cell surface. The second step concerns viral penetration. In the case of enveloped viruses (e.g., HIV, HCV, HSV-1, HSV-2, influenza virus), the virus penetrates cells through the fusion of the viral envelope with the host cell membrane. Envelope glycoproteins are believed to mediate the fusion of the virus envelope with the endosomal membrane of the host cell through a pH-dependent mechanism [33]. In case of non-enveloped viruses, viral particles penetrate the host cells either by translocation of the virion across the host cell membrane or receptor-mediated endocytosis of the virus in clathrin-coated pits, which leads to an accumulation of viruses in cytoplasmic vesicles [34]. The third step in viral replication concerns uncoating, making viral nucleic acids available for transcription to permit multiplication to proceed. It is a complex, virus-dependent process, still not fully understood in some cases. During the following transcription and translation processes (steps four and five), generally known as the replication step, viruses use host cellular machinery to replicate their genome. After generating all the required proteins and genetic material, the next process (assembly, step six) involves bringing together newly formed genomic nucleic acids and structural proteins to form the nucleocapsid of the virus, which are then released from the cell (step seven, release). There are generally three strategies that viruses follow for assembly and release processes. Non-enveloped viruses already exhibit full maturation either in the cytoplasm itself (e.g., picornaviruses) or in the nucleus (e.g., adenoviruses), with disintegration of the cell and release of virions. For enveloped viruses, including the (−) strand RNA viruses and the (+) strand togaviruses and retroviruses, the final maturation of the virion takes place as the virion exits the cell.

Viral proteins are first inserted into the host cell membrane, followed by nucleocapsid binding to the regions of the host cell membranes with these inserted proteins and the formation of a bud into the extracellular space direction. Further cleavage and maturation of proteins may occur after viral extrusion to impart full infectivity on the virion. Next to this mechanism, Herpes viruses, which are enveloped viruses, assemble their nucleocapsids in the nuclei of the infected cell and mature at the inner lamella of the nuclear membrane. Virions accumulate in this region, in the endoplasmic reticulum, and in vesicles protected from the cytoplasm. The release of virions from the cell surface is associated with cytolysis [35].

Any of the multiple steps involved in the viral replication cycle may be exploited as a target for antiviral therapy (Figure 2B). Although early attempts at preventing HIV attachment by soluble CD4 proved unsuccessful, small molecular inhibitors that block the gp120-CD4 interaction are promising alternatives. The efficiency of the fusion inhibitor enfuvirtide (T-20), a FDA-approved synthetic peptide that targets the HIV gp41 envelop protein to prevent fusion, has been demonstrated in a series of clinical trials [36]. Indeed, targeting the early steps of viral entry is an attractive therapeutic strategy, since the site of action of the inhibitor is likely to be extracellular and therefore relatively accessible. The multivalent nature of nanoparticles makes them of particular interest to interfere with the attachment and entry of viruses into host cells, thereby blocking viral entry into the cell.

Inhibition of reverse transcriptase (RT) by substrate analogues (nucleoside and nucleotide RT inhibitors (NRTI)) constitutes another important approach of many antiviral regimes. As NRTIs lack a 3′OH group, once incorporated into the growing complementary DNA strand, they act as chain terminators, bringing reverse transcription to an end [37]. The use of molecules named non-nucleoside RT inhibitors, which result in binding-induced conformational changes that inactivate RT, is another attractive possibility [38]. Other potential targets for drug development include the viral assessor proteins as well as cellular proteins necessary for viral replication [39,40]. A variety of gene therapeutic approaches have been proposed to render CD4 cells resistant to HIV-1 infection [41].

In the following, the different strategies developed for the three enveloped viruses HIV, HCV, and HSV will be outlined in more detail to better understand their modes of action.

## 3. Interaction and Treatment of Different Enveloped Viruses with Nanocarriers

### 3.1. Human Immunodeficiency Virus (HIV)

HIV infects cells of the immune system, destroying these cells as well as the immune system’s ability to fight off the invaders. At the moment, more than 35 million individuals are infected with HIV across the globe, with more than two million new HIV infections being reported every day since the last two years and around the same number of individuals having died due to HIV [42]. With this steady increase in mortality, HIV/acquired immunodeficiency syndrome (AIDS) is currently the fourth-leading disease cause of death, with a scenario projected to get worse in the next decade, mainly in Asia, Africa, and Eastern Europe [43]. Highly active antiretroviral therapy (HAART), a therapeutic approach that uses a cocktail of antiretroviral drugs, has significantly improved the life expectancy of HIV-infected individuals over the years. However, the emergence of drug-resistant viral strains and the need for daily administration of several pills have pointed toward the need for alternative therapeutic approaches.

**Figure 3:**
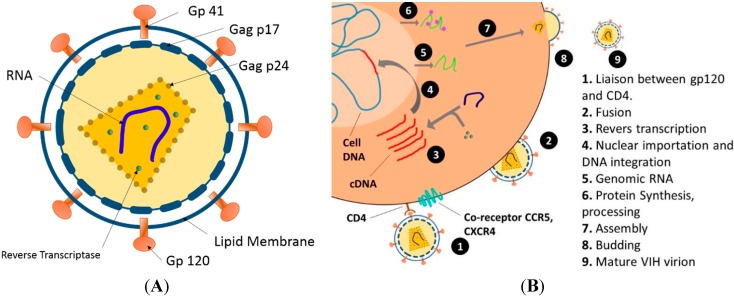
(**A**) Structure of HIV; (**B**) HIV replication cycle [44].

HIV is an enveloped virus, where the outer coating of the virus consists of two layers of lipids with different proteins embedded, forming “spikes” consisting of the outer glycoprotein (gp) 120 and transmembrane gp41. Gp120 is needed to attach to the host cell, and gp41 is critical for the cell fusion process (Figure 3A). The viral core, which is usually bullet-shaped, contains the viral capsule protein p24, which surrounds two identical stands of RNA and the enzymes (reverse transcriptase, integrase, and protease) needed for HIV replication. The size of an HIV virus particle is about 100–150 nm in diameter.

The replication cycle of HIV-1 is a complex, multistep process. It is believed that infection begins with an HIV particle encountering CD4+ cells (cells carrying a CD4 surface molecule (cluster designation 4)). One or more of the virus’s gp120 molecules bind tightly to CD4 molecules on the cell’s surface, which results in a conformational change in the gp120 molecule, allowing it to bind to a second molecule on the cell surface known as a co-receptor [44]. Following gp120 binding to CD4, a cellular receptor, and a subsequent interaction with CCR5 or CXCR4 co-receptors, a conformational change of gp41 leads to membrane fusion and delivery of the capside to the cytoplasm. The RNA is reverse-transcribed into complementary DNA, which is converted to a double-stranded DNA and integrated into the cellular genome. The integrated DNA is transcribed to full-length viral RNA and a number of spliced mRNA transcripts. Transcription and translation, performed by the cellular machinery, result in the synthesis of viral proteins that together with viral RNA are transported to the site of virus particle assembly at the cell membrane, where the virus gains access to the extracellular milieu upon budding events.

HIV/AIDS is currently treated with a combination of 25 antiretroviral drugs that are divided into six classes according to their interference with the HIV life-cycle: fusion/entry inhibitors, integrase inhibitors, protease inhibitors, non-nucleoside reverse transcriptase inhibitors, nucleoside analog reverse transcriptase inhibitors, and multidrug combination products [45]. To overcome the drawbacks of these treatments, particularly low efficacy and therapeutic selectivity, different nanostructures have been proposed over the years as prophylactic agents against HIV [43].

Some of the first attempts to use nanoparticles against HIV-1 were those by Elechiguerra *et al.* using silver nanoparticles (Ag NPs), shown to undergo a size-dependent interaction with HIV-1 [30]. Only particles within the range of 1–10 nm were able to bind to the virus. The precise mechanism of action is not completely understood, but several studies point towards a direct interaction of Ag NPs with surface glycoproteins and interference with the binding and fusion events of viral penetration into susceptible cells [46]. In the case of poly(*N*-vinyl-2-pyrrolidone) (PVP)-coated Ag NPs, the interaction sites were found to be the sulfur-bearing residues of the gp120 glycoprotein knobs. The concentration of PVP-coated Ag NPs at which infectivity was inhibited by 50% (IC_50_) ranged from 440–910 µg/mL. The fact that silver ions exert antiviral effects was not the cause of action, as inhibition of HIV-1 needed a 12-times-higher concentration than Ag NPs. PVP-coated Ag NPs have been thus proposed as a potential topical vaginal microbicide to prevent HIV-1 transmission [47].

**Figure 4:**
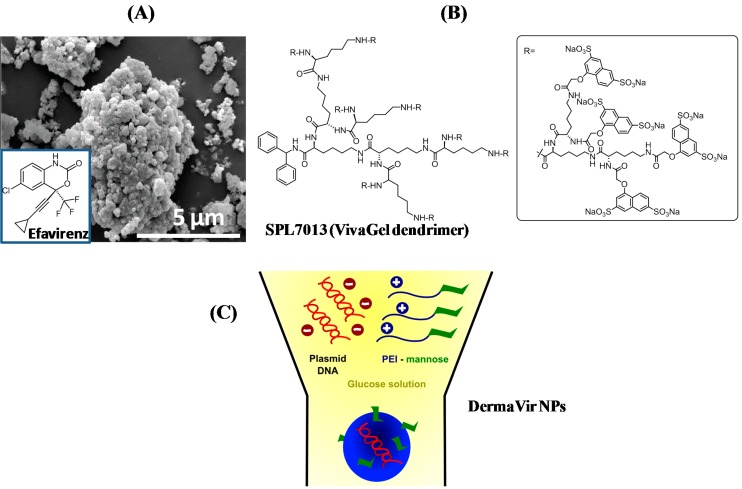
(**A**) SEM image of efavirenz-loaded albumin nanoparticles (reprint with permission from Ref. [57]); (**B**) Chemical structure of VivaGel (SPL 7013) dendrimer; (**C**) Chemical formulation of DermaVir and administration [60].

Other particles have shown the ability to improve the efficiency, solubility, stability, and permeability of anti-HIV drugs (Table 1), through encapsulation of the therapeutics into the nanostructures. Various biodegradable and non-biodegradable polymeric, dendrimeric, lipid, and liposomal delivery systems have been explored to protect HIV-antigens and HIV drugs against extra and intracellular degradation and increase their immunogenicity [11,48,49,50,51,52,53,54,55,56]. The formulation of albumin nanoparticles with efavirenz was recently proposed by Jenita *et al.* as a antiretroviral therapeutic (Figure 4A). Efavirenz is a non-nucleoside reverse transcriptase inhibitor for HIV-1. The formulated particles had a size of 250 nm and exhibited an entrapment efficiency between 45%–72%; they increased efavirenz delivery into various organs by several folds of magnitude in comparison with the free drug [57].

Cationic poly(lactide-coglycolide) (PLG) microparticles modified with gag and env pDNA by surface adsorption were shown to be have substantial potential in the induction of immune responses [58]. The therapeutics applied as a topical microbicide, consisting of l-lysine dendrimers formulated as a carbomer gel (VivaGel, Starpharma), C [10,49,59]. The mechanism of action is based on the poly-lysine branches of the dendrimers, which are terminally derivatized with naphthalene disulfonate groups, responsible for the direct interaction with HIV envelop glycoproteins (Figure 4B). DermaVir differs from other therapeutics by applying multiple targeting elements to achieve active targeting of dendritic cells (Figure 4C). The polymer and the pDNA together form a pathogen-like NP. The surface of the DermaVir NPs contains sugar residues that are important for the uptake by antigen-presenting cells. Inside the cells, the polymer protects the pDNA from endosomal degradation and facilitates the delivery of pDNA into the nucleus. These steps are essential for potent expression of DNA-encoded antigens. It is topically administered with a new medical device (DermaPrep) that supports loading the NPs into the epidermis.

**Figure 5:**
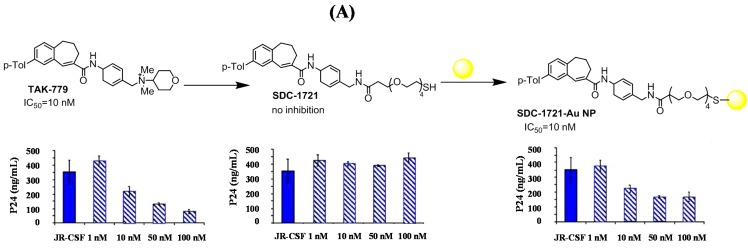

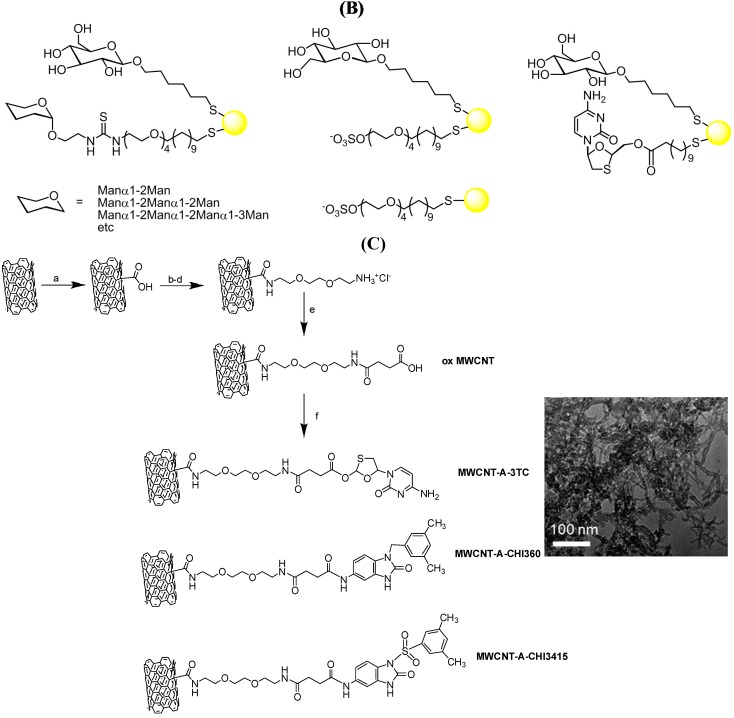
(**A**) Formation of SDC-1721-modified gold nanostructures via ligand exchange reaction, together with results of inhibition assay (reprint with permission from Ref. [62]); (**B**) Different gold-based anti-HIV scaffolds [65,66,67,68]; (**C**) Synthesis of different MWCNT structures investigated as anti-HIV agents together with TEM image of MWCNT-A-CHI360 [62] (reprint with permission from Ref. [70]).

Gold nanoparticles (Au NPs) have shown promising potential as effective inhibitors of HIV fusion [61,62]. The toxicological consequences of using Au NPs are highly related to their properties, such as size and shape, composition, charge, and surface functionality. Appropriate surface coating can thus reduce toxicity to a minimal level and allows better internalization and selective targeting [63]. These nanostructures also have the advantage of allowing the formation of multivalent therapeutics, transforming a weakly binding and biologically inactive molecule into a multivalent conjugate that effectively inhibits HIV-1 fusion to human cells. Bowman *et al.* were the first to demonstrate the inhibition of HIV fusion with multivalent Au NPs of 2 nm in diameter [62]. Mercaptobenzoic acid-modified Au NPs were conjugated to SDC-1721, a derivative of TAK-779, a known CCR5 antagonist, through ligand exchange (Figure 5A). SDC-1721 is indeed lacking the quaternary ammonium salt known to be essential for high-affinity binding and effective HIV-fusion inhibition [64]. By simple conjugation onto Au NPs in a multivalent manner, the low-affinity of biologically inactive SDC-1721 can be overcome and switched to a therapeutically active molecule. To evaluate the antiviral activity of the nanoparticles, phytohemagglutine-stimulated peripheral blood mononuclear cells were infected with the CCR5-tropic HIV-1 clone JR-CSF in the presence and absence of the test compounds. The HIV-1 capsid p24 antigen was measured by ELISA (*enzyme-linked immunosorbent assay*) (Figure 5A). While TAK-779 inhibited HIV-1 replication with an IC_50_ of 10 nM, SDC-1721 did not inhibit viral replication. However SDC-1721 conjugated to Au NPs at an average ratio of 12/1 showed an IC_50_ of 10 nM. The group of Penadés proposed the usefulness of carbohydrate-coated Au NPs as carriers for different structures related to the HIV envelop (Figure 5B) in several articles [65,66,67,68]. The inhibition of the DC-SIGN-mediated HIV-1 trans-infection of human T-cells was inhibited with Au NPs coated with oligomannoses of the gp120 [66]. Sulfated ligands were shown to interfere with the adhesion/fusion of HIV during entry [67]. Multifunctional Au NPs, with glucose and an antiviral prodrug, were recently proposed as potential anti-HIV candidates Please confirm that meaning of sentence is retained [63]. The drugs were released from the particle scaffold in acidic conditions and inhibited viral replication with IC_50_ of 8 µM. Surface-engineered Au nanorods were also found to be a promising DNA vaccine adjuvant for HIV-1 treatment [69].

Most recently, fullerenes and their derivatives, possessing antiviral activity, have been employed as anti-HIV agents [70,71]. Carbon nanotubes offer potential advantages over other nanoparticles, including their ability to cross cellular membranes and shuttle drugs into various types of cells. The multimodal conjugation of carbon nanotubes allows the insertion of more than one type of functional group to the nanotube surface, which may be a key property that establishes their superiority over other agents. Iannazzo *et al.* recently described the antiviral potential of highly hydrophilic and dispersible carboxylated multi-walled carbon nanotubes with incorporated antiretroviral drugs (benimidazolones, CHI360, and CHI415), newly synthesized agents belonging to a series of active non-nucleoside reverse transcriptase inhibitors and lamivudine, a known antiretroviral nucleoside agent that is currently used [70] (Figure 5C). It was found that oxidized MWCNT (Figure 5C). It was found that oxidized multiwall carbon nanotubes (MWCNT) and MWCNT-C-CHI360 exhibited IC_50_ values of 11.43 µg/mL and 4.6 µg/mL, respectively [70]. Analysis of the data suggested that carboxyl groups and, in general, hydrophilic end groups promote electrostatic and hydrogen bond interactions with the amino acid residues of reverse transcriptase involved in the viral cycle.

### 3.2. Hepatitis C Virus (HCV)

**Figure 6:**
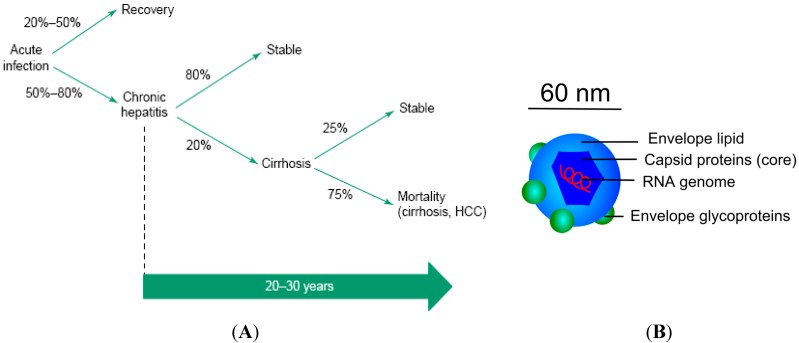
(**A**) Schematic illustration of natural history of HCV infection. HCV infection starts with acute infection and chronic hepatitis can lead to cirrhosis and mortality [74]; (**B**) Structure of hepatitis C virus.

Though HCV was identified first in 1989 [72], its impact on public health has only become known in the past few years. HCV is a major cause of chronic liver diseases leading to fibrosis, cirrhosis, and hepatocellular carcinoma over the course of several years [73]. Humans are natural hosts for HCV and the virus is generally transmitted via blood through medical and surgical procedures, intravenous drug administration, and/or blood transfusion. According to the consensus panel of the National Institute of Health (NIH), HCV infection starts with acute hepatitis in which 20%–80% is self-resolving. In 50%–80% cases, HCV infection develops into a chronic disease, causing hepatitis, cirrhosis (in ~10%–20% of cases after 10–20 years), and hepatocellular carcinoma (HCC) (Figure 6A).

HCV is an envelope virus of 40–60 nm in size with positive-stranded RNA genome (Figure 6B). HCV genotypes are identified from the phylogenetic analysis of full-length or partial sequences of HCV strains, isolated in various regions of the world. The strains include six main groups and various subtypes or subclades [74]. Currently used standard therapies are based on pegylated interferon and ribavirin (Table 1), which inhibit the viral replication step by targeting protease and reverse transcriptase. However, due to the fact of the existence of the genetic diversity during replication in the host, the development of drug-resistant virus mutants started causing significant restricted efficiency of the current anti-HCV drugs for the development of anti-HCV drugs [75]. Some of the new therapeutics targeting the HCV entry process and their stages of development are summarized in Table 2. Some new challenges have to be taken into consideration when designing new antiviral strategies. Current clinical models and HCV-infected patients have, for example, demonstrated that the virus has developed multiple strategies to escape host-neutralizing immune responses during the viral entry process. These include evasion from neutralizing antibodies and viral spread by cell-cell transmission [76]. Well-conserved receptors and co-receptors are now considered to be more promising targets to overcome the high variability of the viral envelope proteins. In addition, the blockage of CD81, SR-B1, and claudin 1 with monoclonal antibodies or small molecules is now considered an efficient way to prevent HCV infection in a genotype-independent manner [77,78,79]. Another way of preventing HCV is to target envelope glycoproteins using small compounds [79], which has, however, been found to be genotype-specific. Blocking the interaction between the highly glycosylated virus envelop and its receptors is considered, as these glycans play major roles in protein folding, HCV entry, and protection of the virus from antibody-dependent neutralization [80,81]. Natural lectins that specifically target glycans of the glycoprotein envelope have been revealed to successfully inhibit the early stage of HCV entry [80,82,83]. However, expenses on the large-scale production and purification of these protein-based anti-viral agents, along with their stability issues and susceptibility to be cleaved by proteolytic enzymes, make them less preferable for eventual use.

**Table 2:** New anti-hepatitis C virus (anti-HCV) compounds targeting viral entry and their stage of development [76].

Target	Examples of Compounds	Stage of Development
HCV E1E2	Neutralizing antibodies	
	Polyclonal HCV IgG (civacir)	Phase II
	HCV-Ab^XTL^68	Phase II
	Human mAB AR3	Mouse model
	CBH and HC antibodies	Cell culture
	IGH antibodies	Cell culture
	AP33	Cell culture
	3/11	Cell culture
	Fab e137	Cell culture
	mAbs 1:7 and A8	Cell culture
	HCV1 and HCV 95-2	Cell culture
	Heparin + HS analogues	Cell culture
	Lectins	Cell culture
HCV particle	Anti-apoEmAb	Cell culture
SR-BI	Anti-SR-BI pAb and mAb	Cell culture
	ITX5061	Cell culture
	Serum amyloid A	Cell culture
CD81	Anti-CD81 mAb	Cell culture, mouse model
	Imidazole-based compounds	Cell culture
CLDN1	Anti-CLDN1 pAb and mAb	Cell culture
Internalization/fusion	PS-ON	Mouse model
	Arbidol	Cell culture
	Chloroquine	Cell culture
	Silymarin	Cell culture

Abbreviations: CLDN = claudin; mAb = monoclonal antibody; pAb = polyclonal antibodies; SR-BI = scavenger receptor class B type I; PS-ON = phosphorothioate oligonucleotides.

**Figure 7:**
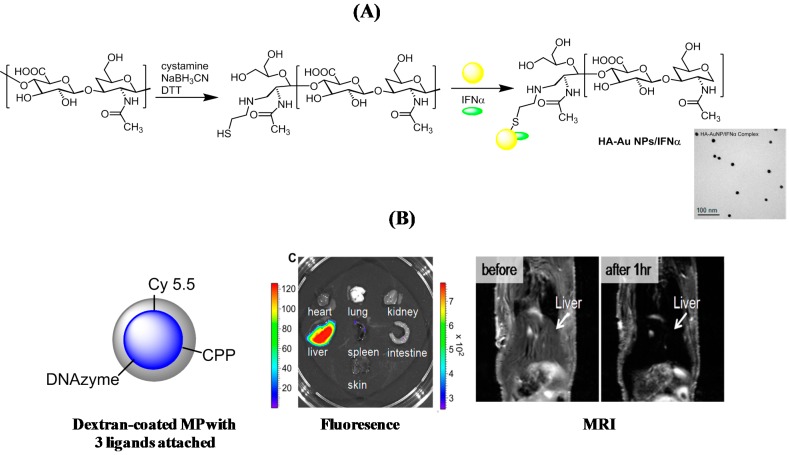
(**A**) Schematic representation for the preparation of thiol-ended HA and the formation of HA-Au NPs/IFNα nanostructures together with TEM image of the gold complex [87]; (**B**) A magnetic nanostructure linked to synthetic DNAzyme, Cy5.5, and a cell penetrating peptide (CPP), (left; fluorescence images of the extracted organs showing the particle accumulation in the liver (middle), T2-weighted MR images of a mouse before and after 1 h injection of the magnetic nanostructure (right) with accumulation in the liver (reprint permission from Ref. [88]).

Nanostructures might also be an alternative to some of the proposed compounds. However, the amount of research being performed in this area is still rather limited. Cationic liposomes [84,85], as well lipid nanosomes [86] with incorporated siRNA, have proven their potential to inhibit HCV gene expression *in vivo*. One of the first attempts for the treatment of HCV infections with nanostructures was that of Lee and co-workers [87]. A target-specific hyaluronic acid (HA)-interferon α (IFnα) Au conjugate was proposed as an alternative to PEGylated IFNα such as PEGASYS and PEG-Intron (Figure 7A). The multifunctional Au nanostructures were prepared by chemical binding of thiolated HA and physical binding of IFNα. According to antiproliferation tests in Daudi cells, the nanostructures showed a comparable biological activity to PEG-Intron with a highly enhanced stability in human serum. The HA-Au NPs/IFNα complex significantly enhanced expression of 2,5′-oligoadenylate synthetase 1 (OAS1) for innate immune responses to viral infections in the liver tissue. This was much higher than those observed using IFNα, PEG-Intron, and AuNP/IFNα, showing the synergetic effect of Ha with IFNα. In the study carried out by Wang *et al.*, Au NPs were utilized to design a nanozyme that mimics the RNA-cleavage function of the active RNA-induced silencing complex (RISC), the cellular machinery that mediates RNA interference pathways [33]. This nanozyme decreases 99% of HCV RNA levels in mice.

Dextran-coated magnetic iron oxide nanoparticles conjugated to a synthetic DNAzyme, targeting a gene of interest, a near-infrared fluorescent dye (Cy5.5) for tracking the particles *in vitro* and *in vivo* using florescence imaging, and a cell-penetrating peptide (CPP) that aids in membrane translocation, were fabricated and used for the treatment of HCV infection (Figure 7B) [88]. The approach was based on the synthetic DNAzyme inducing knockdown of hepatitis C virus (HCV) gene NS3, which encodes helicase and protease essential for the virus replication. Fluorescence imaging showed that the particles accumulated primarily in the liver (Figure 7B). The iron oxide core was, in addition, used for tracking via non-invasive magnetic resonance imaging (MRI) of the nanostructures (Figure 7B), showing accumulation in the liver as seen with fluorescence imaging. Such nanostructures have a high potential for further therapeutic applications and treatment of HCV.

**Figure 8:**
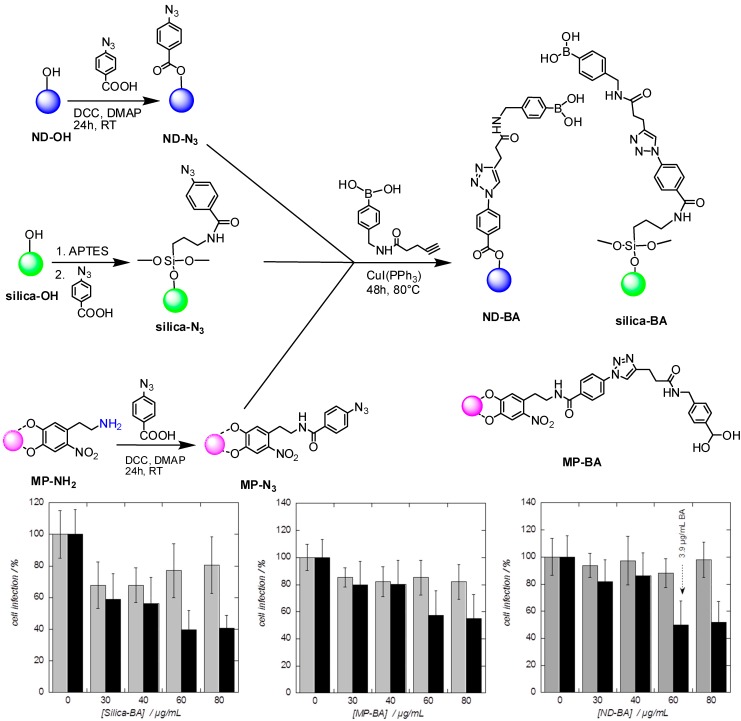

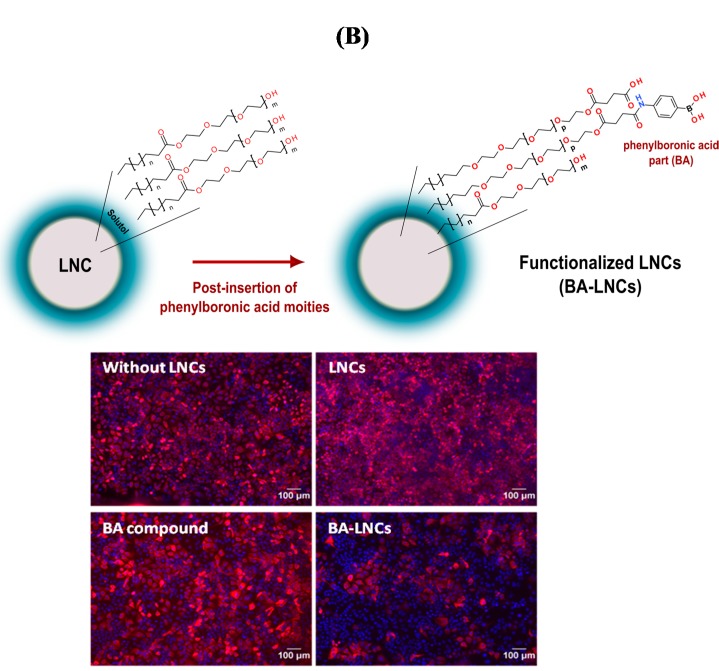
(**A**) Schematic illustration of the fabrication of boronic acid-modified nanoparticles and effect of particle concentration on the entry inhibition activity; (**B**) Formulation of boronic acid-modified lipid nanocapsules (BA-LNCs) together with HCV infection tests on the Huh 7 cell culture: Fluorescent image (10×) of the Huh 7 target cells after incubation with modified JFH1 virus for 48 h, incubation in the presence of the blank LNCs (80 µg/mL), BA compound (concentration of boronic acid moieties = 15 µM), and BA-LNCs (concentration of boronic acid moieties = 12.4 µM), along with absence of nanocapsules (red color and blue color correspond to infected cells and nucleus, respectively) [89,90].

While these previous nanoparticles-based studies were focused on targeting the replication of the virus, our group recently showed the possibility to inhibit HCV entry to hepatocytes using boronic-acid modified nanostructures [89,90]. Indeed, among the handful of molecules reported to show HCV viral entry inhibitory activity are included a variety of glycan-recognizing proteins (GBPs or lectins) [91]. The molecular basis of the virucidal activity of lectins has been tied to their affinity for selective binding of glycans characteristic of viral surfaces. Those such as cyanovirin-N and griffithsin have been shown conclusively to owe their activity to their ability to interact with high-mannose glycans present on the HCV envelope glycoprotein [83,92]. While early successes in the design of artificial lectins were won at the cost of structural complexity [93,94], other so-called “pseudo-lectins” feature much simpler structures and are more accessible synthetically [95,96,97]. Among these are boronic acid-based compounds. Boronic acid-based pseudo-lectins (or “borono-lectins”) benefit from a number of advantages over their natural counterparts, including being more drug-like, stable, and inexpensive to produce and purify, as well as not being mitogenic. The primary mode of action of such pseudo-lectins is the ability of boronic acid moieties to form, selectively and reversibly, the corresponding tetravalent boronate di-ester cyclic complexes with either 1,2- or 1,3 *cis*-diols, such as those typically present in saccharides [98]. The rate of formation/dissociation of such phenylboronate di-ester complexes is, in addition, pH-dependent and occurs most rapidly when boron is tetrahedral, which is favored at higher pH values [99,100]. In addition, when multiple copies of an appropriate boronic acid derivative are grafted onto a scaffold such as a nanoparticle, the affinity for a given monosaccharide or glycoprotein is significantly increased when compared with that of the starting monomer. The pseudo-lectins investigated as HCV entry inhibitors included iron-, silica-, and diamond-derived nanoparticles all featuring surface-attached boronic acid moieties (Figure 8A). A convenient general protocol for attaching multiple *para*-substituted phenylboronic acid moieties to all particles has been used by first modifying each particle-type with surface 4-azidobenzoic ester function. These azide-terminated nanoparticles could be conveniently reacted with 4-[1-oxo-4-pentyn-1-yl) amino]phenyl-boronic acid units to fabricate the targeted pseudo-lectin-like nanoparticles. An assay that measures the potential of cell culture-derived JFH1 virus to infect hepatocytes demonstrated that several of these newly synthesized boronic acid-modified nanoparticles were able to reduce viral entry effectively. While having established the general concept of HCV entry inhibition, a major obstacle to any further development of these nanostructures as viral entry inhibitors was their moderate maximal inhibition potential. Lipid nanocapsules (LNCs) functionalized with amphiphilic boronic acid (BA) moieties through their post-insertion into the semi-rigid shell of the LNCs proved to be far superior as HCV entry inhibitors. These second generation particles (BA-LNCs) (Figure 8B) were found to prevent HCV infection in the micromolar range (IC_50_ = 5.4 µM of BA moieties), whereas the corresponding BA monomer showed no significant effects even at the highest analyzed concentration (20 µM). The new BA-LNCs are the most promising boronolectin-based HCV entry inhibitors reported to date and are thus seen to show great promise in the development of a pseudolectin-based therapeutic agent.

### 3.3. Herpes Simplex Virus

**Figure 9:**
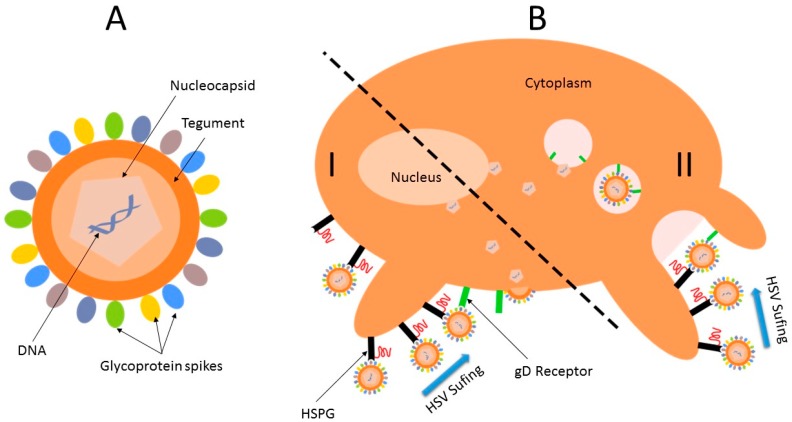
(**A**) HSV virion; (**B**) Entry mode of HSV into cells [105].

Herpes Simplex viruses (HSV) always existed throughout human history. It is transmitted among humans through physical contact and, after the initial infection, the virus remains latent in neurons. Due to this asymptomatic infected period called latency, many infected individuals are unaware of their infection. Both herpes simplex virus type 1 and type 2 (HSV-1 and HSV-2) are large, fragile viruses, infecting 60%–90% of adults worldwide. HSV-1 is associated mainly with infections of the mouth, pharynx, face, eye, and central nervous system, while HSV-2 causes infections of the anogenital area, although an increased proportion of genital infections are caused by HSV-1. In immuno-compromised individuals, babies, and patients with HIV, HSV-1 can be a life-threatening disease. Like HCV and HIV, HSV possesses an envelope (Figure 9A). The central core, containing the linear double-stranded DNA (from 120 to 230 kbp) genome, is enclosed by an envelope of isohedral capsids, which is surrounded by a lipid bilayer envelope that accommodates 11–12 viral glycoproteins. The envelop diameter ranges from 170–200 nm and contains an array of protruding glycoprotein spikes, making the full diameter of the viron about 225 nm on average. Each viron contains about 600–750 spikes with variable packing densities.

HSV entry into host cells is the first and most critical step in viral pathogeneses [101] and is one of the most comprehensively understood routes among members of the Herpesviridae family (Figure 9B). The penetration of HSV-1 into the host cell involves at least five viral envelop glycoproteins (gC, gB, gD, gH, gL), together with three classes of host cell membrane receptors (herpes virus entry mediator, nectin-1, nectin-2, and cell surface glycosaminoglycans, preferentially herpan sulfate) [102]. Thus, viral membrane proteins bind, prior to fusion with plasma or endocytotic membranes, to cellular receptors. During this attachment phase, gC and gB interact independently with cellular heparan sulfate in an irreversible manner. The affinity of the binding of gC to heparin sulfate is in the order of 10^−8^ M and is considered to be the major binding interaction during attachment. Indeed, when gB and gC are absent, viral binding to the cell surface is severely reduced. Accordingly, in cells that are deficient in heparan sulfate, a ubiquitous constitute of cell plasma membranes and extracellular matrices, mediating various physiological processes, a drastic reduction in susceptibility to viral infections results. Herpes envelope gB and gC glycoproteins bind heparin sulfate, whereas a third glycoprotein, gD, has a specific interaction with 3-*O*-sulfated heparin sulfate. These properties make heparin sulfate highly attractive to generate targeted nanoparticles via anti-heparan sulfate peptide conjugation [103,104]. After fusion, nucleocapsides containing the viral genome are released into the cytoplasm and transported to nuclear pores. Viral DNA is then released into the nucleus. The viral replication step is short (18–24 h) and results in cytolysis.

**Figure 10:**
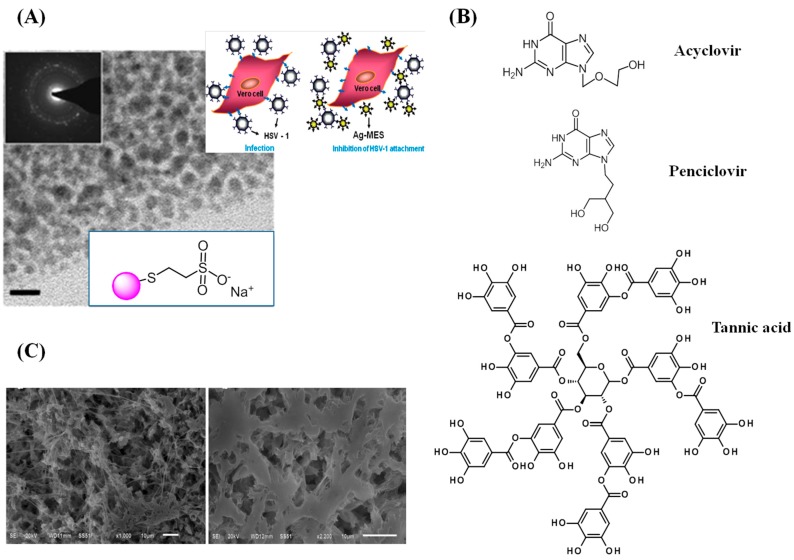
(**A**) Mercaptoethane sulfate-modified Ag NPs together with TEM image and mode of inhibition (reprint with permission from Ref. [108]); (**B**) Chemical structures of anti-herpes drugs acyclovir, penciclovir, and tannic acid; (**C**) Microporous polycaprolactone matrix loaded with 7% acyclovir for controlled delivery of antiviral agents (repring permission from Ref. [111]).

Most anti-herpes drugs are targeted against the viral DNA polymerase. Acyclovir, a guanosine analogue (Figure 10B), has been the most successful and important clinical drug for the prophylactic and therapeutic treatment of HSV infections and is considered as the “golden standard” in HSV therapy. However, extensive use of this therapeutic has led to the emergence of HSV-resistant virus strains, particularly in immuno-compromised patients. This has led to the search for alternative drugs as well as for therapies that interfere with viral attachment at different levels. Potential viral and cellular targets for the development of anti-Herpes drugs are currently based on the inhibition of DNA polymerase using small organic molecules, as well as antiviral analogues (nucleoside-based molecules) or natural molecules (e.g., oxyresveratrol), compounds that target the primase-helicase complex and inhibit ATPase activity, the delivery of siRNA for down-regulating specific proteins, the inhibition of viral attachment and penetration based on sulfated and sulfonated polysaccharides or tannins or through the inhibition of the secretory form of HSV-1 glycoprotein B (gB1s) or through mimicing the amine terminal portion of the recombinant gB1s by polylysine rich molecules [2,106]. The potential of nanoparticles-based anti-Herpes drugs has been a more recent topic of research with only a few structures currently proposed. Baram-Pinto *et al.* described the potential of Ag and Au NPs capped with sulfonate functions, formed through a sonochemical approach, for the inhibition of HSV-1 infections [107,108] (Figure 10A). The mercaptoethanesulfonate (MES)-decorated Ag NPs of 4 nm in size mimic heparan sulfate (HS) on the host cell [108]. A concentration of 40 µg/mL was able to block the virus attachment to the cells. In the same way, sulfonate-decorated Au NPs inhibited, at the same concentration, the entrance of the virus into the cells and prevented the cell-to-cell spread of HSV-1 [107]. The mode of action proposed of MES-Ag NPs is that they serve as multivalent inhibitors that mimic HS on the host cell membrane and therefore effectively inhibit HSV by blocking viral attachment to the cell (Figure 10A). A similar approach using partially negatively charged ZnO micro-nano structures (MNSs) was reported by Mishra *et al.* to trap the HSV-1 at low concentrations (10 µg/mL) *in vitro* via the ionic interactions with the positively charged viral envelope of glycoproteins in order to prevent virus-cell interactions [109]. The ZnO MNSs were capped with nanoscopic filopodia-like spikes which mimic the cellular filopodial HS structure with a partial negative charge due to the oxygen vacancies present on them. Later, Antoine *et al.* also showed that ZnO tetrapod structures significantly block HSV-2 entry into target cells [110].

Ag NPs modified with tannic acid (Figure 10B) were reported by Orlowski *et al.* to be capable of reducing HSV-2 infectivity both *in vitro* and *in vivo* [111]. Tannins, high molecular weight polyphenolic structures, are capable of forming insoluble complexes with proteins. The high affinity of tannins for HSV glycoproteins prevents the attachment and the entry of virions into the host cell. The multivalent character of the tannic acid-modified Ag NPs is responsible for the observed anti-viral effects. The size of the particles was found to be crucial for the overall anti-viral character. Taking into account the HSV structure consisting of glycoprotein spikes with an average center-to-center spacing of 9–13 nm, tannic acid-modified Ag NPs of 33 nm in diameter proved to be the best-performing ones, due to an optimal size and loading effect.

Most reports on anti-Herpes therapeutics are based on the encapsulation of efficient antiviral agents such as acyclovir, peniclovir, oxyresveratrol, *etc.* (Figure 10B) [112,113,114,115]. However, these synthetic molecules present a low oral bioavailability due to slow absorption in the gastrointestinal tract. For these reasons, new approaches such as polymeric formulations and nano-encapsulations with polymers have been developed. Polycaprolactone (PCL) matrices containing acyclovir, produced by cooling suspensions of the drug in PCL solution followed by solvent extraction, were proposed by Asvadi *et al.* for the controlled delivery of acyclovir for the treatment and prevention of HSV infections [115]. Another example of the delivery system was based on cyclodextrin-based nanosponges loaded with acyclovir [116]. The acyclovir loading in 400 nm spherical carboxylated cyclodextrin-based nanosponges could reach about 70% (*w*/*w*) and showed enhanced antiviral efficiency against a clinical isolate of HSV-1.

Microemulsions, isotropic systems of oil, water, and surfactant, frequently in combination with a co-surfactant containing droplets in the nanosize range, have in particular shown to be of potential interest for delivering HSV drugs. Acyclovir was thus introduced in microemulsions using 2.5% transcutol as permeation enhancer for *in vivo* studies [25]. Female mice infected by HSV-1 were treated by a single application of this formulation and the development of herpetic skin lesions was totally suppressed in 24 h. Two other studies showed the development of microemulsion-based topical formulations of penciclovir [113,114]. Yu *et al.* showed that a formulation containing oleic acid, Cremophor EL, ethanol, and water formed stable microemulsions and increased the permeation of penciclovir about 3.5 times through excised mouse skins when compared to commercial creams [115].

Recently, oxyresveratrol, a natural compound abundantly derived from a traditional Thai plant, appeared highly effective in delaying the development of skin lesions [117].

**Figure 11:**
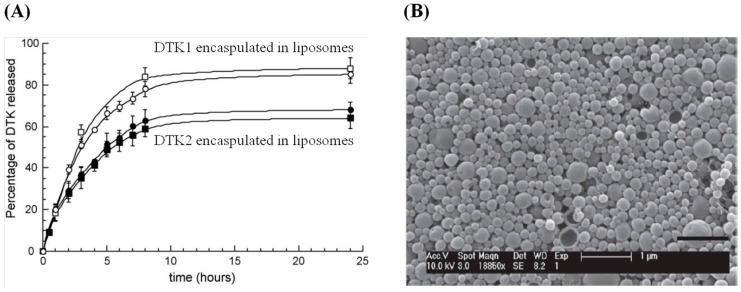
(**A**) Release profile of DTK1 and DTK2 encapsulated in liposomes; (**B**) SEM image of PLGA-siRNA particles and *in vitro* nectin mRNA knockdown comparing si-nectin lipoplex (reprint with permission from Ref. [122]).

Cortesi *et al.* demonstrated that cationic liposomes, containing the secretory form of HSV-1 glycoprotein B (gB1s) or two related polylysine-rich peptides (DTK1 and DTK2), allowed the inhibition of HSV-1 infection [118]. This approach permitted overcoming the rapid drug loss, the long time release of DTK1 and DTK2 (Figure 11A), and the poor corneal permeability of the active agents for opthalmic delivery. Even though both vaccines did not show total protection, rabbits were sheltered against HSV lethal infection and viral reactivation. In another study, a topical liposomal gel of idoxuridine (IDU) showed an increase of skin retention of IDU due to its entrapment in the liposomal carriers [119].

In recent years, specific proteins involved in HSV infection were able to be down-regulated using short interfering RNA (siRNA) macromolecules encapsulated into polymer structures [120,121]. Steinbach *et al.* demonstrated that the intravaginal administration of nanoparticles made of poly(lactic-co-glycolic acid) (PLGA)-encapsulating siRNA is effective in the prevention of genital HSV-2 infection in mice [122] (Figure 11B). The particles were designed to interfere with HSV-2 infection by siRNA-mediated knockdown of nectin, a host cell protein.

Erythrocytes, more commonly known as red blood cells (RBC), possess long a circulation half-life time, are non-immunogenic, and have been investigated in recent years to target anti-viral agents, especially for HSV-1[123]. The secretory form of HSV-1 glycoprotein B (gB1s) was linked to RBC for the immunization of C57BL/6 mice via a biotin-avidin-biotin bridge. After the treatment, mice were protected against HSV-1 infection, and induced a similar or higher anti-HSV antibody response than a mixture of free gB1s and adjuvants. As RBC may deliver nucleotides, Rossi *et al.* reported in 2001 on the encapsulation of an antiviral agent based on a 9-(2-phosphonylmethoxyethyl)adenine (PMEA) molecule bound by a phosphate bridge [124]. This strong and selective nucleotide analogue (Bis-PMEA) was found to be active against HIV-1 and HSV-1. Bis-PMEA-loaded erythrocytes were still found 10 days after phagocytosis in macrophages and showed a good protection from the HSV-1 (85%) as well as HIV-1 (95%) infection.

## 4. Conclusions

There is a continuous and vital need for new antiviral therapeutics and approaches to confront the emergence of drug resistance and different secondary effects due to long-term treatment. While many bacterial infections have shown to be successfully treated with antibiotics, different antiviral agents have entered the market much later. Indeed, in contrast to bacteria, a virus is a small micro-organism that can only reproduce inside a living host cell. Treatment is thus much more complicated, with the consequence that the most serious communicable diseases known to medical science are viral in origin. One also has to keep in mind that antiviral drugs are currently only effective against a few viral diseases, such as influenza, herpes, hepatitis B and C, and HIV—but research is ongoing. With the continuous emergence of new viral infections, the search for viral therapeutics is thus an endless task. Nanotechnology has demonstrated to be well placed to bring some important changes in the overall scenario. As outlined in this review, over the last decade, various nanomaterials have been explored and evaluated for their prophylactic and therapeutic activity against different viruses. The high potencies of Ag NPs due to their antibacterial and antiviral activities have been investigated at the beginning. While they indeed proved to have significant antiviral effects against several viruses, such as HIV, herpes simplex, hepatitis, respiratory syncytial, and influenza, the adverse effect of Ag NPs is their high cytotoxicity to many mammalian cells. The use of smartly designed dendrimeric, polymeric, and multivalent Au NPs showed to be better adapted for this purpose. Some of these nanomaterials, when combined with classical antiviral agents, improved the solubility and stability of the therapeutics, and allowed controlled long-term release of the drug. This is of great benefit to reduce the number of doses and to improve patients’ adherence. However, at the moment, only one nanoparticulate-based product, VivaGel, a dendrimer with the ability to inhibit HIV-1, is under evaluation in Phase 1 clinical trials [125]. While the future of nanoparticles-based therapy is promising, there are still many challenges and barriers to achieve its full potential. Several aspects still need to be considered and optimized for a successful translation of nanomaterials from the laboratory to the clinical setting. In fact, most of the studies using anti-viral nanostructures are limited to either cell culture or *in vivo* models. Beside the dendrimeric VivaGel (SPL 7013), no commercial pharmaceutical nanocarrieres are on the market. Compared to anti-viral agents on the market (Table 1), the use of nanomaterials as “pharmaceutical nanocarriers” is still in its infancy and time will show if the currently developed approaches and concepts might be successful. The debate about the toxic nature of nanoparticles and their effect on human health upon prolonged exposure is still ongoing. Nanotoxicology is still a rather new discipline, requiring further development in terms of models and assays. Without a deeper knowledge of the impact of nanoparticles on health, large-scale production and application in various fields will not be fully considered. From a scientific point, receptor-based nanoparticles are one of the ways of achieving a secured administration of nanoparticles as anti-viral agents. The integration of functional groups and units onto the surface of nanoparticles allows for effectively targeting specific sites of viral infections. This allows reducing drug-related toxicity in other tissues and increasing the therapeutic dose at the target site, making receptor-based designed nanoparticles of the utmost importance in clinical scenarios. What became clear, however, over the years, is that only a multidisciplinary consortium, including chemists, physicists, materials scientists, and biologists, as well as medical doctors, will be able to answer some of the outstanding questions related to antiviral agents and their use for human treatment.
